# Multidimensional Assessment of Electroencephalography in the Neuromodulation of Disorders of Consciousness

**DOI:** 10.3389/fnins.2022.903703

**Published:** 2022-06-23

**Authors:** Chunyun Zhang, Shuai Han, Zean Li, XinJun Wang, Chuanxiang Lv, Xiangyun Zou, Fulei Zhu, Kang Zhang, Shouyong Lu, Li Bie, Guoyue Lv, Yongkun Guo

**Affiliations:** ^1^Department of Neurosurgery, The First Hospital of Jilin University, Changchun, China; ^2^Department of Neurosurgery, Fifth Affiliated Hospital of Zhengzhou University, Zhengzhou, China; ^3^Department of Pediatrics, Qilu Hospital of Shandong University, Qingdao, China; ^4^Department of Hepatobiliary and Pancreatic Surgery, The First Hospital of Jilin University, Changchun, China; ^5^Henan Engineering Research Center for Prevention and Treatment of Brain Injury, Zhengzhou, China

**Keywords:** transcranial direct current stimulation, disorders of consciousness, brain network, electroencephalography, neuromodulation, minimal consciousness, vegetative state, graphy theory

## Abstract

In the present study, we aimed to elucidate changes in electroencephalography (EEG) metrics during recovery of consciousness and to identify possible clinical markers thereof. More specifically, in order to assess changes in multidimensional EEG metrics during neuromodulation, we performed repeated stimulation using a high-density transcranial direct current stimulation (HD-tDCS) protocol in 42 patients with disorders of consciousness (DOC). Coma Recovery Scale-Revised (CRS-R) scores and EEG metrics [brain network indicators, spectral energy, and normalized spatial complexity (NSC)] were obtained before as well as fourteen days after undergoing HD-tDCS stimulation. CRS-R scores increased in the responders (R +) group after HD-tDCS stimulation. The R + group also showed increased spectral energy in the alpha2 and beta1 bands, mainly at the frontal and parietal electrodes. Increased graphical metrics in the alpha1, alpha2, and beta1 bands combined with increased NSC in the beta2 band in the R + group suggested that improved consciousness was associated with a tendency toward stronger integration in the alpha1 band and greater isolation in the beta2 band. Following this, using NSC as a feature to predict responsiveness through machine learning, which yielded a prediction accuracy of 0.929, demonstrated that the NSC of the alpha and gamma bands at baseline successfully predicted improvement in consciousness. According to our findings reported herein, we conclude that neuromodulation of the posterior lobe can lead to an EEG response related to consciousness in DOC, and that the posterior cortex may be one of the key brain areas involved in the formation or maintenance of consciousness.

## Introduction

Disorders of consciousness (DOC) are states of loss of consciousness that are caused by a range of severe brain injuries (Giacino et al., 2002; Wannez et al., 2017b). DOC lasting longer than 28 days are considered chronic (prolonged) disorders of consciousness (pDOC), which are divided into vegetative state (VS), minimal consciousness state (MCS), and emergence from MCS state (eMCS) presentations. Based on language processing, the MCS entity is additionally divided into minimally conscious state minus (MCS−) and minimally conscious state plus (MCS+) subcategories; MCS- is characterized by visual pursuit and fixation, localization to noxious stimuli, and/or automatic motor responses, whereas in MCS + patients can follow instructions, make understandable verbalizations, and/or communicate intentionally but not functionally (Thibaut et al., 2019).

A widely accepted clinical tool for assessing patients’ level of consciousness (LOC) is the Consciousness Recovery Scale-Revised (CRS-R), which consists of six subscales assessing auditory, visual, motor, oral motor, communication, and arousal states. The total maximum score is 29 points, and the diagnosis is determined by the best assessment of each of the subscales (Wannez et al., 2017a; Kondziella et al., 2020).

In addition, transcranial direct current stimulation (tDCS) is a non-invasive neuromodulation technique that increases neuronal excitability by depolarizing resting potentials (Weber et al., 2014; Lefaucheur et al., 2017; Shou et al., 2021). Previous studies have partially demonstrated that tDCS can also be used as a therapeutic tool to increase the LOC in patients with DOC by stimulating the left dorsolateral prefrontal lobe (DLPFC), though other studies have shown no meaningful benefit of tDCS as a treatment modality (Giacino et al., 2014; Thibaut et al., 2019; Feng et al., 2020). We note that Wu et al. demonstrated that the strength of intrinsic functional connectivity in many brain regions, particularly in the posterior cingulate cortex and precuneus, correlated statistically significantly with LOC and recovery outcomes in patients with DOC. Thus, the posterior cortex could be used as a potential stimulation target (Wu et al., 2015).

Neuroimaging allows for the objective recording of central nervous system damage after acquired brain injury, providing additional information about the diagnosis, prognosis, and recovery process in regard to consciousness, and serving as a potential marker for novel therapeutic interventions. Compared to positron emission tomography (PET) and functional magnetic resonance imaging (fMRI), electroencephalography (EEG) is an ideal tool for monitoring brain function because it can record the spontaneous and rhythmic electrical activity of neuronal populations at the millisecond scale in order to effectively study topological features in regard to brain function (Laureys and Schiff, 2012; Edlow et al., 2013).

In this study, we chose the posterior cortex as the stimulation target, referring to previous studies, and used HD-tDCS as the stimulation paradigm in order to evaluate the resulting changes in multidimensional EEG metrics (Cai et al., 2019). We aimed to elucidate changes in EEG metrics during recovery of consciousness and to identify possible clinical markers thereof. We consider these analyses to be exploratory and therefore proposed two hypotheses. Firstly, we hypothesized that the use of Pz (i.e., the midline parietal) as a stimulus target would allow for large-scale functional network modulation and a shift toward regularized networks. Secondly, we attempted to build a predictive model to detect responsiveness in patients with DOC.

## Materials and Methods

### Patients

Forty-five patients with pDOC were recruited from the Departments of Neurosurgery at the Fifth Affiliated Hospital of Zhengzhou University and the Zhengzhou Central Hospital (Zhengzhou University) between October 2016 and October 2020. Patients were classified as presenting in a VS or MCS according to the CRS-R scale, using an awakening stimulation protocol if necessary in order to determine these classifications.

Patient inclusion criteria were as follows: (1) no scalp lesions or intracranial metal implants; (2) no history of neurological or psychiatric disorders; (3) no acute illness or episodes of chronic illness; and (4) patients with LOC lasting for more than 28 days.

Exclusion criteria were as follows: (1) the presence of intracranial anterior and posterior lobe lesions, (2) meaningful fluctuations in LOC in the week prior to stimulation with HD-tDCS; (3) the previous presence of a pacemaker, aneurysm clips, or other metallic devices; and (4) incomplete cranial bone.

Study participants were enrolled for 2 weeks of HD-tDCS stimulation. Forty-five patients completed this HD-tDCS stimulation. The epidemiological characteristics of the enrolled patients are provided in Supplementary Table 1.

**FIGURE 1:**
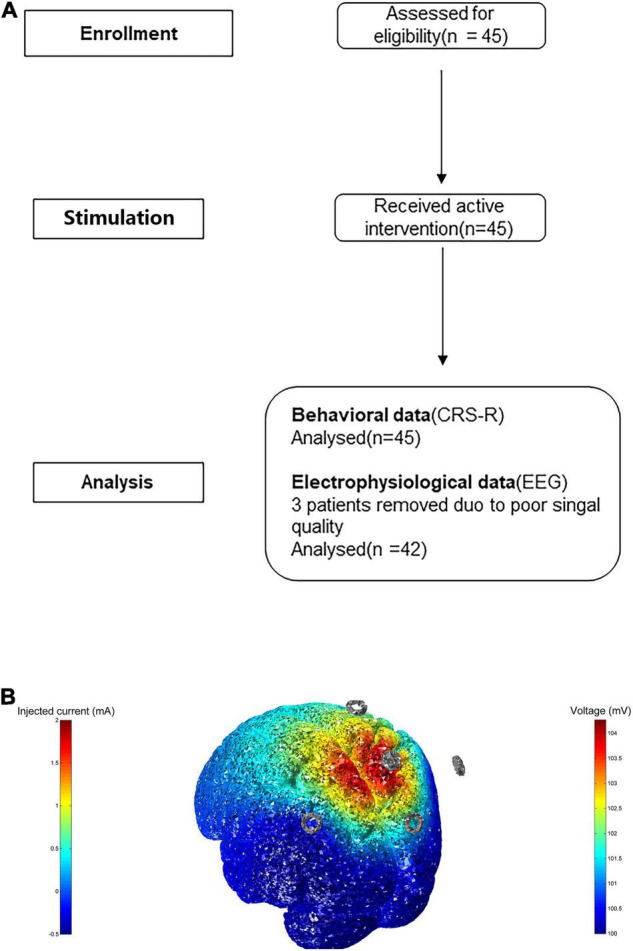
**(A)** Flowchart of study screening. **(B)** The model of transcranial electric stimulation. Here we inject 2 mA at electrode Pz, and cathode is at CPz, POz, P3, P4, displaying 3D rendering of the computed voltage and electric field distribution.

The study protocol was approved by the Ethics Committees of the Fifth Affiliated Hospital of Zhengzhou University (approval number: KY2020024) and Zhengzhou Central Hospital, Zhengzhou University (approval number: 201614), and informed consent was obtained from all patients’ families and caregivers. This word was conducted in accordance with the principles of the Declaration of Helsinki. The flowchart depicting the study process is shown in Figure 1A.

### High-Density Transcranial Direct Current Stimulation Protocol

Stimulation was performed in the Pz region using a transcranial electrical stimulator (Soterix Medical Inc., New York, NY, United States) with a current of 2 mA for 20 min; cathodic electrodes were located at the CPz (centro-parietal), POz (midline posterior), P3 (left parietal), and P4 (right parietal) electrodes. The current was applied to the cortex through an Ag-AgCl ring electrode. The anode was placed at Pz according to the international 10/20 system. Four cathode electrodes, placed symmetrically at a distance of 3.5 cm around Pz, approximately corresponded to the four electrodes mentioned above (Huang et al., 2018, 2019; Guo et al., 2019). A 14-day period of twice daily stimulation was administered to all patients, in the morning and the afternoon, and any adverse effects of HD-tDCS were monitored and recorded. Due to the lack of individual T1 MRI data, we visualized a 3D rendering of the voltage and electric field distribution on an MNI152 averaged head. This protocol is shown in Figure 1B.

### Outcomes

The focus of the study was the effect of HD-tDCS on subsequent changes in electrophysiological indices and LOC. We highlight that, as mentioned above, any adverse effects were strictly monitored and recorded during the stimulation (Giacino et al., 2004; Gerrard et al., 2014; Di et al., 2017).

### Electroencephalography Recording and Processing

The EEG equipment used herein included a Nicolet EEG amplifier (Natus Neurology Inc., Middleton, WI, United States), and the EEG cap used the 32 electrodes of the international standard 10–20 system. The skin impedance of the electrodes was kept below 5 kΩ before the experiment. The recording lasted for 10 min. For some patients, the CRS-R arousal stimulation protocol was applied immediately before the EEG recording, and the patient remained awake during EEG acquisition. If a patient closed his or her eyes for more than a few seconds or showed sleep features (i.e., sleep spindles or K complex waves in the EEG), the EEG recording was terminated and the data was discarded. Following this, the CRS-R awakening protocol (with EEG acquisition) was repeated. Patients did not receive any enteral and parenteral nutrition before the 30 min of EEG recording.

Offline preprocessing was performed using EEGLAB running in a MATLAB environment (MathWorks, Natick, MA, United States) with self-programmed scripts (Delorme and Makeig, 2004). Firstly, the EEG data were bandpass filtered (0.5–40 Hz), and the notched filter (48–52 Hz) was applied to the EEG signal to eliminate the effect of the alternating current [AC; 50 Hz intermediate frequency (IF) signal]. Next, the sampling rate was downsampled to 500 Hz and the data were re-referred using an average amplitude. The EEG signal was then divided into 5 s repetition-free segments. In the next step, the bad segments were removed through manual detection and the bad channels were complemented by spherical interpolation. The remaining EEG data were evaluated using an independent components analysis. Topography, weights in the epochs, and the spectrum were used to detect and remove eye blinking/movement components. Data with <80% retention was considered invalid (Straudi et al., 2019; Zhang et al., 2020). In order to overcome the volume-conduction of the EEG signals, we analyzed current source density (CSD) before calculating the EEG features (Kayser and Tenke, 2006; Bai et al., 2019).

#### Resting-State Electroencephalography Analysis

Resting EEG was analyzed using spectral energy, functional network parameters based on graph theory, and normalized spatial complexity (NSC). The EEG signal was divided into seven frequency bands: delta (0.5–4 Hz), theta (4–8 Hz), alpha1 (8–10.5 Hz), alpha2 (10.5–13 Hz), beta1 (13–20 Hz), beta2 (20–30 Hz), and gamma (30–40 Hz).

#### Spectral Power Analysis

Previous studies have demonstrated that spectral energy is correlated with patients’ LOC. Spectral power in the 0.25 Hz range was calculated using Welch’s method for the Fourier decomposition of the data periods. In each channel, the power values of the seven standard bands were converted to the relative percentage contribution of the total power of all bands.

#### Graph Theory

The brain network was constructed based on patients’ resting-state EEG signals. The network nodes were defined as the electrodes. The connected edge of the network was defined as the phase-locked value (PLV) of the EEG signal between the two electrodes. The PLV used only phase information in the calculation process, which can separate the phase and amplitude components of the EEG signal and avoid the effect of data amplitude. The phase-based connectivity analysis relied mainly on the phase difference of the two individual electrode signals. The PLV calculated by this method ranges from 0 to 1 and does not require parameter selection (Nunez et al., 1997, 1999; Lachaux et al., 1999). The constructed brain network was then quantitatively analyzed using graph theory (Iakovidou, 2017; Sporns, 2018). Based on the Gretna toolbox, we calculated the following properties: global efficiency, local efficiency, small-world properties, and node attributes (node clustering coefficients) (Wang et al., 2015). Prior to topological characterization, a thresholding procedure was typically applied to exclude the confounding effects of spurious relationships in regard to interregional connectivity matrices. We chose network sparsity as the thresholding method employed herein. See Supplementary Material for more information about the thresholding procedure.

The term clustering coefficient refers to the coefficient of the degree of information aggregation in a node; the larger the clustering coefficient, the stronger the ability of that node to transmit information. Global efficiency is used to measure the functional integration of the network and the global transmission capacity of the network. The local efficiency of the network indicates the efficiency of information transfer in regard to sub-networks. The small-world property shows the optimal balance between network separation and integration. Prior to topological characterization, the method of sparsity thresholding was invoked to calculate the area under the curve (AUC) for different parameters since there is currently no explicit method for selecting individual thresholds. In order to make the network parameter calculation independent of the network size, 100 times the random network size was used. The random network was obtained by randomly resampling the nodes and connected edges of the original network (Mancini et al., 2016; Iakovidou, 2017).

#### Normalized Spatial Complexity

The spatial complexity of neural signals, traditionally quantified using omega complexity, was inversely proportional to the level of functional connectivity in the region of interest (ROI), thus providing a new approach to functional connectivity analysis. The higher complexity value represents a more concentrated level of connectivity and a more complex amount of information transmitted within the network. However, the omega complexity measure was sensitive to the number of neural signal time series. Jia et al. (2018) proposed the use of principal component analysis (PCA) and normalized entropy to effectively estimate the spatial complexity of neural signals, defined as NSC, which could reflect the global functional connectivity of brain signals and overcome the limitations inherent to omega complexity.

Firstly, temporal PCA was performed on the EEG signals in order to derive 30 principal components and eigenvalue spectra. Secondly, in order to evaluate the relative contribution of each principal component to the total variance, the eigenvalues of the principal components were normalized to the unit sum. Finally, the whole-brain spatial complexity of seven individual frequency bands was calculated according to the method presented in the study conducted by Gao et al. (2017) and Jia et al. (2018). The NSC calculated above obtains values in an interval ranging from 0 to 1. The minimum value of 0 implies that the EEG signals of all scalp channels consist of only one principal component or spatial pattern, predicting a maximum functional link between all channels. A maximum value of 1 indicates that the total data variance is evenly distributed over all 30 principal components, predicting either the greatest spatial complexity or the lowest functional linkage across the brain.

### Machine Learning for the Response to Transcranial Direct Current Stimulation

Traditional modeling methods, such as linear and logistic regression, place strict limitations on the number of features. Herein, two groups were formed according to whether patients presented with new consciousness representations. A machine learning structure was designed to classify responders (R +) and non-responders (R-). We selected seven bands representing whole-brain spatial complexity as features and behavioral scale responsiveness parameters as labels in order to build a model. Nest structured machine learning was used to construct a binary classification model (Shen et al., 2017; Bai et al., 2021). The first layer consists of an inner loop for parameter estimation, and the second layer consists of an outer loop for prediction evaluation. Data were kept independent between the inner and outer loops. Different models and parameters were tested within the inner loop, and the best model and best parameters were selected. In the outer loop, the selected model and parameters were applied to the independent data and prediction was performed. We evaluated model performance using accuracy evaluations, the confusion matrix, and the AUC. We assessed the significance of the predictive power using permutation tests (1,000 iterations).

The choice of modeling algorithms included support vector machine (SVM), linear discriminant analysis (LDA), random forest (RF), and K-nearest neighbor (KNN) algorithms (Pedregosa et al., 2011). The details of these methodologies are provided in the Supplementary Material.

## Statistical Analyses

Statistical analyses of behavioral data and EEG indicators were performed using R statistical software (The R Project for Statistical Computing, Vienna, Austria). A Wilcoxon rank-sum test (WR-test) was used for behavioral data. For spectral energy, global properties derived through graph theory, and spatial complexity, paired *t*-tests were used to test the statistical significance of the differences caused by stimulation. For nodal properties derived through graph theory, including node clustering coefficients, paired *t*-tests were used to test the statistical significance of the differences caused by stimulation at the electrode level. A false discovery rate (FDR) correction (*Q* = 0.05) was used to correct the resulting *p*-values after conducting comparative evaluations of the involved electrodes (Bai et al., 2019; Martens et al., 2019).

## Results

### Behavioral Response After High-Density Transcranial Direct Current Stimulation

**FIGURE 2:**
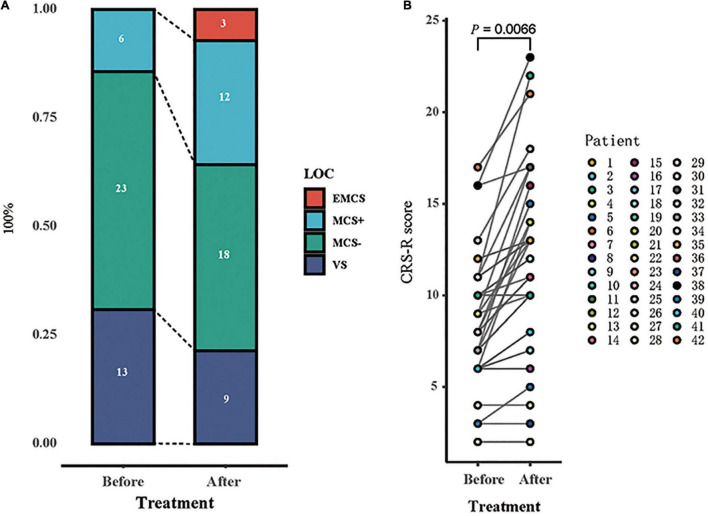
Behavioral response to tDCS. **(A)** The proportion of each state of consciousness, before and after tDCS showed an increase in the higher states of consciousness [emergence from minimally conscious state (eMCS) and minimally conscious state “plus” (MCS +)], at the expense of lower states of consciousness [vegetative state (VS) and MCS “minus” (MCS−)]. **(B)** Individual patients’ CRS-R scores before and after tDCS are represented with the number of patients and their state (symbols). Significant difference in scores before and after stimulation is found (*p* = 0.0066).

After 14 days of HD-tDCS stimulation, the total CRS-R score increased in 32/42 patients compared with the score at baseline (from 8.26 ± 3.52 to 11.07 ± 5.16, *p* = 0.007). Patients were divided into two groups based on whether they presented with new conscious representations: responders (R +) and non-responders (R-); the EEG indicators of the two groups were analyzed separately. The behavioral response to HD-tDCS is shown in Figure 2.

### Spectral Power

**FIGURE 3:**
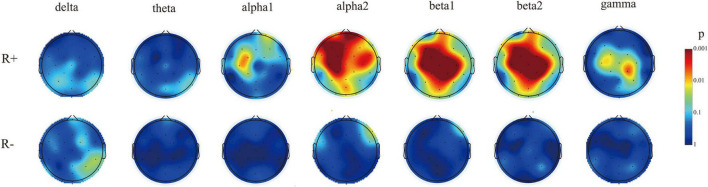
The electrodes with a significant increase in spectral energy after stimulation compared to pre-stimulation in two groups of patients. The electrodes with increased energy in the R + group were mainly concentrated in the alpha2 and beta1 bands, while the R− group only had increased energy in the alpha2 and beta1 bands of F8 electrodes.

In the R + group, by comparing the spectral energy of seven bands at pre- and post-stimulation, we found that the energy of some electrodes in the alpha1, alpha2, beta1, beta2, and gamma bands increased (mainly in the alpha 2 and beta1 bands); the main increase was detected in the frontal and parietal electrodes. The electrodes with increased energy were as follows: alpha1 band: Fpz, F3, F4, C3, FC1, and CP1; alpha2 band: FP1, FP2, F3, F4, C3, C4, P3, O1, F7, F8, T7, Fz, Pz, FC5, Cz, FC1, FC2, FC6, CP1, CP2, CP6, Fpz, FOz, and Oz; beta1 band: FP2, F3, F4, C3, C4, P3, P4, C4, P3, P4, O1, O2, F7, Fz, Pz, FC5, Cz, FC1, FC2, FC6, CP1, CP2, CP6, Fpz, POz, and Oz; and beta2 band: F3, C3, C4, P4, Fz, Pz, Cz, FC1, FC2, CP1, CP2, and POz; gamma band: C3, P4, Cz, FC1, FC2, CP1, and CP2. No electrodes with increased energy were found in the delta and theta bands. In the R- group, in evaluations of the spectral energy of the seven bands at T1 as compared with that at T0, we found that the F8 channel showed an increase in energy only in the alpha2 and beta1 bands, while no energy increase channels were seen in the alpha1, beta2, delta, theta, or gamma bands. The above results are depicted in Figure 3.

### Graph Theory

Regarding the global properties in the R + group, the area under the global efficiency curve in the alpha1 band increased after stimulation compared with that before stimulation (*p* = 0.047), the area under the local efficiency curve increased compared with that before stimulation (*p* = 0.033), and the small world property sigma increased after stimulation compared with that before stimulation (*p* = 0.026). In contrast, in the R- group, the above three indices did not change at the level of statistical significance, with resulting *p*-values of 0.679, 0.640, and 0.924, respectively. In the beta1 band, the small-world property sigma increased in the R + group after stimulation as compared to pre-stimulation (*p* = 0.011), while there was no statistically significant difference in the R- group (*p* = 0.392). In the theta band, the global efficiency of the R + group did not differ at the level of statistical significance before and after stimulation (*p* = 0.285), while that in the R- group increased after stimulation as compared to pre-stimulation (*p* = 0.039). In addition, the local efficiency of the R + group decreased after stimulation compared to that before stimulation (*p* = 0.034), while the local efficiency in the R- group did not differ statistically significantly before and after stimulation (*p* = 0.181).

**FIGURE 4:**
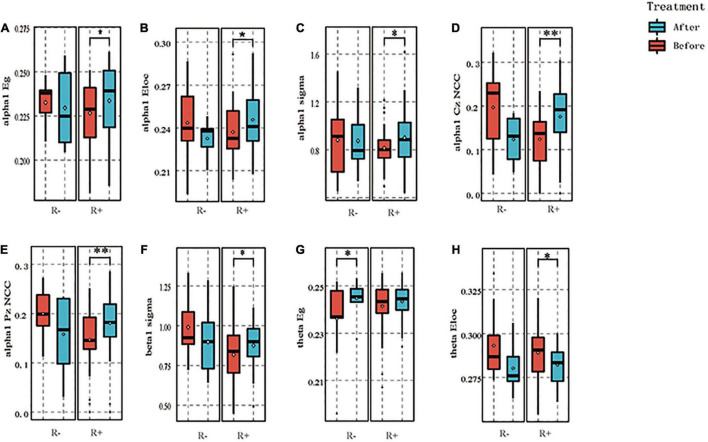
The graph theory results for both groups. α1 band: in the R + group, the area under the global efficiency curve increased after stimulation compared with before, *p* = 0.047 **(A)**, the area under the local efficiency curve increased compared with before, *p* = 0.033 **(B)**, and its small-world index sigma increased after stimulation, *p* = 0.026 **(C)**, whereas in the R- group, the above three indexes did not change significantly, *p* = 0.679, 0.640, and 0.924, respectively. The cluster coefficient of Cz nodes in the R + group increased compared with pre- stimulation, *p* = 0.003, while that of the R- group decreased after stimulation, *p* = 0.031 **(D)**, the cluster coefficient of Pz nodes in the R + group increased compared with that before stimulation, *p* = 0.003, while that of the R- group did not differ significantly, *p* = 0.130 **(E)**. Beta1 band: in the R + group, the small-world attribute sigma was elevated after stimulation compared to before, *p* = 0.011, while there was no significant difference in the R- group, *p* = 0.392 **(F)**. theta band: no significant difference was seen in the R + group between before and after stimulation for global efficiency, *p* = 0.285, while the R- group was elevated after stimulation compared to before, *p* = 0.039 **(G)**; local efficiency in the R + group decreased after stimulation compared to before stimulation for local efficiency, *p* = 0.034; while no significant difference was seen in the R- group before and after stimulation, *p* = 0.181 **(H)**. *p < 0.05, **p < 0.01.

In terms of node properties, the cluster coefficient for Cz nodes in the R + group in the α1 band increased compared to pre-stimulation (*p* = 0.003), while that in the R- group decreased (*p* = 0.031); the cluster coefficient for Pz nodes in the R + group increased compared to pre-stimulation (*p* = 0.003), while the cluster coefficient in th R- group did not differ statistically significantly before and after stimulation (*p* = 0.130). The above results are shown in Figure 4.

### Normalized Spatial Complexity

**FIGURE 5:**
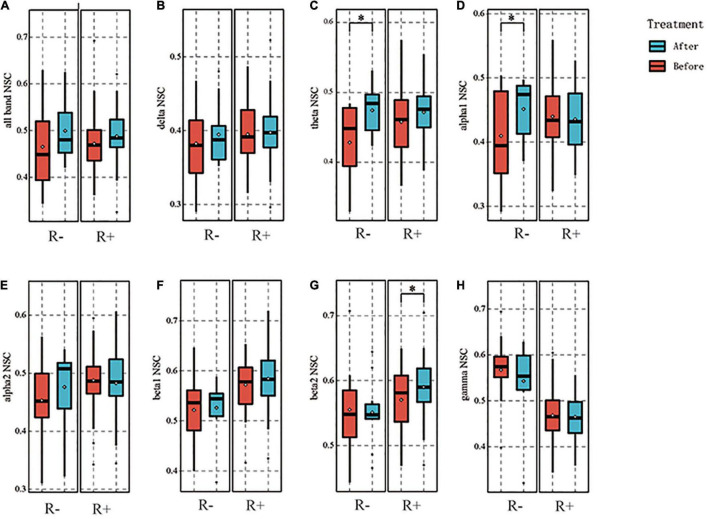
Results of normalized spatial complexity (NSC) for each frequency band. For the R- and R + group, the NSC of the all frequency band was not significantly different between before and after stimulation, *p* = 0.206, 0.084 **(A)**. Delta band did not differ between before and after stimulation, *p* = 0.708, 0.448, respectively **(B)**. Theta band was not significantly different for the R + group, *p* = 0.071; the R- group was higher after stimulation than before stimulation, *p* = 0.024. **(C)**. In the alpha1 band, no significant difference was found in the R + group between before and after stimulation, *p* = 0.608, and the R- group increased after stimulation, *p* = 0.035 **(D)**. Alpha2 band also showed no significant difference between before and after stimulation for the two groups, *p* = 0.504, 0.127, respectively **(E)**. In the beta1 band, no significant difference was found between the two groups before and after stimulation, *p* = 0.370 and 0.756, respectively **(F)**. In the beta2 band, the R + group showed an increase compared to the pre-stimulation, *p* = 0.030, and the R- group showed no difference, *p* = 0.849 **(G)**. In the gamma band, no difference was observed between the two groups, *p* = 0.122 and 0.468, respectively **(H)**. *p < 0.05.

We additionally calculated the full-band NSC and found that no statistically significant differences were observed between the R + and R- groups (*p* = 0.206, 0.084). Moreover, we calculated the NSC for each frequency band. No difference was found in the delta band before and after stimulation in two groups (*p* = 0.708, 0.448). In the theta band, no statistically significant difference was seen in the R + group before and after stimulation (*p* = 0.071), whereas the NSC in the R- group increased after stimulation compared to pre-stimulation (*p* = 0.024). In the alpha1 band, no statistically significant difference was observed for the R + group (*p* = 0.608), while the R- group showed an increase in NSC compared to pre-stimulation (*p* = 0.035). In the alpha2 band, no statistically significant difference was observed for the two groups before and after stimulation (*p* = 0.504, 0.127). In the beta2 band, there was an increase in the R + group compared to pre-stimulation (*p* = 0.030), while no difference was observed in the R- group (*p* = 0.849). In the gamma band, no difference was observed for either of the groups (*p* = 0.122, 0.468). The above results are shown in Figure 5.

### Predictive Model

To predict the responsiveness of patients after 2 weeks of the evaluated tDCS stimulation protocol, we chose the NSC of seven frequency bands at the baseline as features for model training and used SVM (linear kernel), SVM (Gaussian kernel), RF, LDA, and KNN methods, respectively. The SVM (linear kernel) was found to work best. The area under the receiver operating characteristic curve was 0.9 and its accuracy was 0.929. Sensitivity and specificity were 100 and 70%, respectively.

**FIGURE 6:**
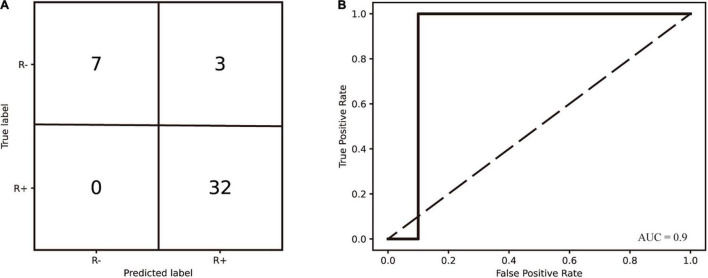
**(A)** The confusion matrix of diagnosed consciousness classification generated by the SVM (linear kernel). The classifier was trained on the NSC metrics derived from the Resting-EEG. **(B)** Receiver operating characteristic curves for SVM (linear kernel) prediction models.

We calculated the *p*-value of the permutation test as the proportion of sampled permutations greater than or equal to the true prediction labels. The number of iterations was 1,000. A *p*-value (<0.001) was obtained by the abovementioned permutation test. The seven band weights were 0.139, 2.563, 6.707, 4.150, 2.445, 1.457, and 11.602. The alpha and gamma bands had the highest weights, indicating that alpha and gamma band NSC was most important in predicting treatment responsiveness. The accuracy, sensitivity, specificity, AUC, and permutation test *p*-values of the evaluated models are shown in Table 1. The receiver operating characteristic curve and confusion matrix of the SVM are shown in Figure 6.

**TABLE 1:** Performance of ML approaches for the estimation of predicted accuracy in patients to tDCS protocol.

Model	Predicted accuracy	AUC	Sensitivity	Specificity	*p*-value (Permutation test)
K-nearest neighbor	0.881	0.750	1.0	0.5	<0.001
Linear discriminant analysis	0.905	0.888	0.969	0.7	<0.001
Random forest	0.857	0.842	0.969	0.5	<0.001
Support vector classifier (linear kernel)	0.929	0.900	1.0	0.7	<0.001

*MLmachine-learning; tDCS transcranial direct current stimulation.*

## Discussion

In this study, we divided patients into R + (32 patients) and R- (10 patients) groups according to the observed improvement in their CRS-R scores. The CRS-R showed that some patients presenting in a VS improved after the long duration stimulation, while some patients presenting in an MCS showed no change in responsiveness. Response heterogeneity in HD-tDCS suggests that behavioral scales alone do not accurately assess stimulus effects and predict patient responsiveness, which may be partially attributable to underlying functional network deficits.

We innovatively chose Pz as the anodic target and the surrounding four electrodes as the cathodic targets. With this stimulation protocol, we obtained a stimulation electric field more concentrated in the posterior cortex. To investigate the potential modulatory effects of the stimulation protocol on neural activity in patients with DOC, this study analyzed three quantitative metrics in regard to resting-state EEG: power spectrum, graph theory, and NSC. Finally, a prediction model was built using the NSC of individual frequency bands as features to assess the DOC response.

In the R + group, the spectral power of multiple electrodes in the alpha2 and beta1 bands was increased. These electrodes were mainly placed in the frontal and parietal regions. In terms of brain networks, we chose the PLV to build a functional brain network and calculated graph theory metrics based on the brain network to assess the stimulation effect. In regard to global metrics, we found that the global efficiency, local efficiency, and small-world property sigma in the alpha1 band of the R + group were higher than before stimulation, as was the small-world property sigma in the beta1 band, suggesting that the patient’s brain network had changed to a regular brain network rather than an irregular network after the administered stimulation. In contrast, for the R- group, there was no difference detected before and after the stimulation. In the theta band, local efficiency decreased after stimulation for the R + group, while no difference was seen in global efficiency and small-world attribute sigma; in contrast, for the R- group, global efficiency increased compared to before stimulation. Similarly, in terms of node properties, the alpha1 frequency band Cz and Pz node clustering coefficients increased in the R + group compared to pre-treatment, while no differences were seen in the R- group. The prediction model suggested that the NSC of the alpha and gamma bands best determines patient responsiveness to the evaluated stimulation protocol.

In our study, we stimulated a posterior parietal lobe stimulation target using HD-tDCS. The posterior parietal lobe is thought to play an important role in the generation of consciousness. In recent years, many consciousness studies have debated the role of the prefrontal and posterior parietal cortex in the formation of consciousness; however, a growing number of studies suggests that the posterior cortex may be a more decisive brain region for consciousness, as consciousness can be preserved even if most of the patient’s prefrontal cortex is removed (Laureys and Schiff, 2012; Koch et al., 2016; Boly et al., 2017; Mashour, 2018). It is particularly important to identify the possible origin of consciousness as a stimulus target and to clarify the network changes induced by the modulation of this target in order to explore the mechanisms underlying HD-DCS and consciousness generation. This study demonstrated that the evaluated stimulation protocol could modulate spectral power in the alpha, beta, and gamma bands, mainly through frontal and parietal electrodes.

Moreover, to explore the effect of network modulation, this study conducted a graph-theoretic analysis of the functional network. Our results showed that the protocol could improve the nodal efficiency of Pz and Cz in the alpha1 band and improve the information transfer properties of the nodes; all of this may improve the global efficiency and local efficiency of the alpha1 band, enable easier mutually connect between brain regions, and enable brain networks to consume less energy in communication. The above changes may transform the brain network to a normal network. This is demonstrated by the increased small-world property of the alpha1 band seen herein. We note that a small-world network that can handle complex tasks and provide near-optimal local and global functional connectivity.

For the R- group, an increase in local efficiency was observed only in the theta band. Moreover, an elevated NSC in the beta2 band could be observed in the R + group; this was inversely proportional to functional connectivity. More specifically, the higher the value, the more information the channel presented and the more intensive the local network connection. Only a few electrodes in the beta2 band showed an increase in spectral power, which may be lateral proof of this concept. All of the above suggests that, in the higher bands, brain network connectivity is concentrated in fewer nodes. This tends to lead to an exchange of information within a local range. In contrast, in the lower bands, there is a tendency for long-distance connectivity of the network. The VS changes to a wide range of cortical functions and network impairments, which tend to disrupt small-world properties more severely (Cai et al., 2020b). We therefore hypothesize that promoting information clustering of network nodes in the higher bands and restoration of long-range links in the lower bands may be an underlying mechanism for this protocol.

At the same time, the key role of the posterior parietal lobe in the origin of consciousness was also laterally demonstrated. A growing number of studies have shown that many brain disorders involve changes in brain network features, such as integration and dissociation, that are often based on a modular approach. Cai et al. found that the brain network of patients with DOC exhibited more integration and less dissociation compared to controls (Cai et al., 2020a). Chen et al. (2013) demonstrated that, when moving from VS to MCS, the aggregation coefficient in the alpha network had an increasing effect that tended to have an increased likelihood of generating positive clinical outcomes. In addition, the increased Pz and Cz clustering coefficients in the alpha1 band detected in this study indicate an increase in local information processing capacity. Moreover, according to the previous study, the configuration of brain networks follows two major principles: one tends to minimize overall wiring costs and facilitate module formation (isolation), and the other promotes efficient global communication (integration). These two principles compete, and the trade-off between the principles determines the efficient organization of the network (Chen et al., 2013; Avena-Koenigsberger et al., 2015; Sporns and Betzel, 2016; van den Heuvel and Sporns, 2019; Cai et al., 2020a). Considering that random networks exhibit higher integration and lower segregation than real networks, patients in the R + group showed less randomness with higher levels of consciousness, as well as exhibiting more segregation and less integration, which is consistent with the elevated NSC of the beta2 band in the R + group. By including NSC in the model, we obtained a relatively satisfactory result. The alpha and gamma bands had higher weights, indicating that the retention of higher NSC in two bands predicted the recoverability of brain networks in DOC. There is no doubt that combining machine learning with EEG metrics to determine if a patient may respond to a neuromodulation protocol at baseline will benefit clinicians in optimizing protocols.

The natural next step in this field of research includes clarifying how multidimensional EEG metrics evolve during stimulation. If EEG metrics can be detected during the baseline period (i.e., when implementing the tDCS modulation protocol) or before behavioral scale scores improve, EEG multidimensional metric analysis can be used to provide useful feedback informing clinical decisions and optimizing neuromodulation protocols for individual patients. However, determining whether the EEG multidimensional changes observed herein are specific to HD-tDCS or are more generalizable to other brain stimulation protocols is an important issue to resolve in determining the potential clinical utility of these methodologies. Taken together, the current study adds to the preliminary evidence base suggesting that EEG multidimensional metrics are altered in patients who respond to HD-tDCS stimulation. This represents a step toward a better understanding of DOC-related network defects and how these defects are modulated by HD-tDCS modulation.

In addition to the substantial strengths of our study, we acknowledge some limitations to this research. First, a control group was not included in this study, and our findings may simply reflect a more general response of improved consciousness that is confounded by placebo effects, rather than purely reflecting neuromodulation-induced changes. Moreover, medication administration and etiology were not assessed as covariates affecting changes in EEG metrics. Secondly, the small statistical sample size and the imbalance between etiologies (eight strokes, 12 cases of trauma, two cases of hypoxia) and diagnoses (MCS, 29; VS, 13) of the patients included in this study reduces the validity of our study findings. In addition, this study lacked a follow-up evaluation to validate long-term modulatory effects. Although EEG is used a functional neuroimaging tool, some results may be limited by effects of functional connectivity and/or metabolism within brain regions, and we can thus additionally explore mechanisms using multimodal techniques (including fMRI and PET) in order to establish more typical multidimensional quantitative metrics, explore mechanisms, and detect brain imaging biomarkers.

## Conclusion

In conclusion, we used Pz as a target to quantify changes in brain networks in multiple dimensions and combined the derived information with machine learning techniques to build a model with high accuracy that allows for predicting modulatory responsiveness. The changes in multidimensional EEG metrics seen herein may suggest the existence of a common neuromodulatory mechanism and may provide a basis for clinical EEG consciousness improvement as well as for the proposal of neuromodulatory markers. At the same time, we conclude that modulation of the posterior parietal lobe can lead to an EEG response related to consciousness in DOC, and the posterior cortex may be one of the key brain regions involved in the formation and maintenance of consciousness.

## Data Availability Statement

The protocol and the statistical analysis plan are available on request. Because of the privacy regulations of the collaborating hospital, the individual EEG can only be accessible by contacting YG with research application.

## Ethics Statement

The studies involving human participants were reviewed and approved by the Ethics Committee of the Fifth Affiliated Hospital of Zhengzhou University (approval number: KY2020024) and Zhengzhou Central Hospital, Zhengzhou University (approval number: 201614). The patients/participants provided their written informed consent to participate in this study.

## Author Contributions

CZ, LB, and YG contributed to study design. CZ, SH, CL, XW, and XZ contributed to data collection. CZ and ZL contributed to data analysis. ZL, KZ, and FZ contributed to figure and table creation. GL contributed to revision and data analysis. All authors contributed to manuscript writing.

## Conflict of Interest

The authors declare that the research was conducted in the absence of any commercial or financial relationships that could be construed as a potential conflict of interest.

## Publisher’s Note

All claims expressed in this article are solely those of the authors and do not necessarily represent those of their affiliated organizations, or those of the publisher, the editors and the reviewers. Any product that may be evaluated in this article, or claim that may be made by its manufacturer, is not guaranteed or endorsed by the publisher.
