# Supplementary material for: Multidimensional Assessment of Electroencephalography in the Neuromodulation of Disorders of Consciousness
Source: Front Neurosci. 2022 Jun 23;16:903703. doi: 10.3389/fnins.2022.903703 (PMC9260110; doi:10.3389/fnins.2022.903703)
Supplement: Supplementary file 1 [file Data_Sheet_1.DOCX]

**Multidimensional Assessment of** **Electroencephalography In the Neuromodulation of Disorders of Consciousness**

**Supplementary Information**

**Threshold method**

We chose threshold method—sparsity or density threshold. Sparsity is defined as the ratio of the number of actual edges divided by the maximum possible number of edges in a network. For networks with the same number of nodes, the sparsity threshold ensures the same number of edges for each network by applying a subject-specific connectivity strength threshold and therefore allowing an examination of relative network organization. Finally, given the absence of definitive way in selecting a single threshold, researchers can input a range of continuous threshold values to study network properties in GRETNA

**Machine learning**

We selected 7 bands of NSC as features and the responsiveness to tDCS stimulation as labels . Nest structured machine learning was used to construct the binary classification model. To optimize the free parameters, perform a two-layered cross-validation. The first layer is called the inner loop for parameter estimation, and the second layer is called the outer loop for prediction evaluation. Every loop, we divided data into training and testing sets for cross-validation. Data are kept independent between the inner loop and the outer loop. Different models and parameters are tested within the inner loop, and the best model and parameters are selected. In the outer loop, the selected model and parameters are applied to the independent data and prediction is performed. The strategy of inner loop was five-fold validation and the strategy of outer loop was leave-one-out cross-validation (LOOCV). One subject is removed from all data and 44 subjects are used to build the predictive model. Repeat this step in an iterative manner with a different subject left out in each iteration. To perform a five-fold cross-validation in the inner loop, the N-1 subjects are binned into five equal size bins, and for each iteration subjects from four bins are used for training and subjects from the one left-out bin are used for testing. We used the accuracy. We evaluated model performance using accuracy, confusion matrix and area under the curve (AUC). Receiver operating characteristic (ROC) curves plotted the false-positive rates as a function of true-positive rates. The AUC was measured to test the performance of the features in classifying the responseness to tDCS, with a bigger AUC implying a more efficient discrimination of groups. Then we used the permutation testing to assess the prediction significance. Permutation testing was used to generate an empirical null distribution of the test statistic. A large number of possible values of the test statistic are calculated under random rearrangements of the labels for the data. After breaking the true NSC–responsiveness relationship, we use these new label assignments as inputs for building prediction model and store accuracy of every permutation for later use. Repeat this process 10,00 times in order to produce enough samples to estimate the distribution of the test statistic. Calculate the P value of the permutation test as the proportion of sampled permutations that are greater or equal to the true prediction correlation.

Table S1. Characteristics of the patients with disorders of consciousness.

| ID | Gender | Age (years) | Etiology | Diagnosis  at T0 | T0 CRS-R score  (total score/sub-scores) | Diagnosis  at T1 | T1 CRS-R score  (total score/sub-scores) | Group |
| --- | --- | --- | --- | --- | --- | --- | --- | --- |
| 1 | M | 44 | Stroke | MCS- | 9/222102 | MCS- | 10/223102 | R+ |
| 2 | M | 37 | Stroke | VS/UWS | 6/112002 | VS | 6/112002 | R- |
| 3 | M | 62 | Stroke | MCS- | 11/233102 | EMCS | 22/456223 | R+ |
| 4 | M | 48 | Trauma | VS/UWS | 6/112101 | VS | 6/112101 | R- |
| 5 | F | 52 | Stroke | MCS- | 8/222101 | MCS+ | 15/344102 | R+ |
| 6 | F | 65 | Stroke | VS/UWS | 6/112002 | MCS- | 8/122102 | R+ |
| 7 | M | 30 | Stroke | MCS- | 7/122101 | MCS- | 13/333112 | R+ |
| 8 | M | 28 | Trauma | MCS- | 11/232202 | MCS+ | 13/332212 | R+ |
| 9 | M | 51 | Stroke | VS/UWS | 6/212001 | MCS- | 8/222101 | R+ |
| 10 | M | 51 | Trauma | MCS- | 9/115101 | MCS- | 13/235102 | R+ |
| 11 | F | 40 | Anoxia | VS/UWS | 6/112101 | VS | 6/112102 | R- |
| 12 | M | 36 | Stroke | VS/UWS | 6/112101 | VS | 7/112102 | R+ |
| 13 | F | 52 | Trauma | MCS- | 9/222102 | MCS+ | 17/345112 | R+ |
| 14 | F | 60 | Stroke | MCS- | 11/232112 | MCS+ | 17/434213 | R+ |
| 15 | M | 8 | Trauma | MCS- | 7/122101 | MCS+ | 16/335212 | R+ |
| 16 | M | 54 | Trauma | VS/UWS | 6/112101 | MCS- | 6/112101 | R- |
| 17 | F | 51 | Stroke | MCS- | 6/122101 | MCS+ | 14/333212 | R+ |
| 18 | M | 48 | Trauma | VS/UWS | 6/113001 | MCS- | 7/123001 | R+ |
| 19 | M | 52 | Stroke | MCS- | 10/233101 | MCS- | 12/234102 | R+ |
| 20 | M | 38 | Stroke | MCS- | 8/213101 | MCS+ | 14/334112 | R+ |
| 21 | F | 38 | Stroke | MCS- | 8/113201 | MCS- | 11/124202 | R+ |
| 22 | F | 54 | Stroke | MCS- | 7/113101 | MCS- | 10/233101 | R+ |
| 23 | F | 62 | Stroke | MCS- | 6/103101 | MCS- | 8/104102 | R+ |
| 24 | F | 72 | Stroke | MCS+ | 12/323112 | MCS+ | 13/333112 | R+ |
| 25 | M | 54 | Trauma | VS/UWS | 2/001001 | VS | 2/001001 | R- |
| 26 | M | 14 | Trauma | MCS- | 10/232102 | MCS- | 12/233112 | R+ |
| 27 | M | 41 | Stroke | VS/UWS | 4/102001 | VS | 4/102001 | R- |
| 28 | M | 52 | Stroke | VS/UWS | 2/011000 | VS | 2/011000 | R- |
| 29 | M | 55 | Stroke | MCS- | 8/222101 | MCS- | 11/233102 | R+ |
| 30 | M | 63 | Stroke | MCS- | 10/322102 | MCS- | 10/322102 | R- |
| 31 | F | 60 | Stroke | MCS+ | 16/344113 | MCS+ | 17/444113 | R+ |
| 32 | M | 70 | Stroke | MCS+ | 13/324112 | MCS+ | 18/445212 | R+ |
| 33 | F | 66 | Stroke | MCS- | 7/122101 | MCS- | 10/232122 | R+ |
| 34 | M | 67 | Stroke | MCS- | 11/233102 | MCS+ | 13/333112 | R+ |
| 35 | F | 69 | Anoxia | MCS+ | 12/332112 | MCS+ | 13/432112 | R+ |
| 36 | M | 65 | Stroke | MCS- | 10/134101 | MCS- | 11/234101 | R+ |
| 37 | M | 60 | Anoxia | VS/UWS | 3/011001 | VS | 3/011001 | R- |
| 38 | M | 52 | Trauma | MCS+ | 16/444112 | EMCS | 23/456323 | R+ |
| 39 | M | 54 | Trauma | VS/UWS | 3/001101 | VS | 5/002102 | R+ |
| 40 | M | 61 | Stroke | MCS- | 6/121101 | MCS- | 8/122102 | R+ |
| 41 | M | 67 | Trauma | MCS- | 10/233101 | MCS- | 10/233101 | R- |
| 42 | F | 62 | Stroke | MCS+ | 17/345113 | EMCS | 21/456312 | R+ |
|  |  |  |  |  |  |  |  |  |
| Summary  (mean$\pm$1SD) | M: 28  F: 14 | 51.55$\pm$15.3 | Stroke:28  Trauma:12  Anoxia:2 | MCS:29  VS/UWS:13 | 8.26$\pm$3.52 | EMCS:3  MCS:30  VS/UWS:9 | 11.07±5.16 | R+:32  R-:10 |

F - female; M - male; CRS-R - Coma Recovery Scale-Revised; MCS - minimally conscious state; VS/UWS - vegetative state/unresponsive wakefulness syndrome; eMCS emergence from MCS; R+ responder; R- non-responder
